# Retinitis pigmentosa‐1 due to an *RP1* mutation in a consanguineous Iranian family: Report of a novel mutation

**DOI:** 10.1002/ccr3.8666

**Published:** 2024-03-14

**Authors:** Mostafa Neissi, Motahareh Sheikh‐Hosseini, Javad Mohammadi‐Asl

**Affiliations:** ^1^ Department of Genetics Khuzestan Science and Research Branch, Islamic Azad University Ahvaz Iran; ^2^ Department of Genetics Ahvaz Branch, Islamic Azad University Ahvaz Iran; ^3^ Noor‐Gene Genetic Laboratory Ahvaz Iran; ^4^ Pediatric Cell & Gene Therapy Research Center Tehran University of Medical Sciences Tehran Iran; ^5^ Department of Medical Genetics, School of Medicine Ahvaz Jundishapur University of Medical Sciences Ahvaz Iran

**Keywords:** exome‐sequencing, mutation, retinitis pigmentosa, *RP1* gene

## Abstract

**Key Clinical Message:**

The identification of a novel *RP1* gene mutation highlights the importance of precise variant identification for retinitis pigmentosa prognosis and genetic consultations, emphasizing comprehensive genetic analysis for personalized care.

**Abstract:**

Our study unveils a noteworthy association between retinitis pigmentosa‐1 and a newly discovered homozygous mutation (c.5326delC; p.Asp1777Ilefs*32) within the *RP1* gene. This highlights the crucial role of accurate variant identification in not only informing prognosis but also improving genetic consultations and influencing future diagnostic approaches for individuals affected by retinitis pigmentosa.

## 1 INTRODUCTION

Retinitis pigmentosa (RP, MIM 268000) stands as the most prevalent manifestation among retinal dystrophies, distinguished by the progressive degeneration of rod and subsequently cone photoreceptors.^1^, ^2^ The manifestation of symptoms unfolds with night blindness during adolescence, followed by peripheral visual loss in young adulthood, ultimately progressing to central visual loss and, in severe cases, total blindness.^1^, ^2^ The classic triad of ocular changes encompasses optic disc pallor, attenuated retinal vessels, and diffuse pigmentary alterations in the retina. RP, marked by genetic heterogeneity, boasts over 601 identified chromosomal loci, with 53 resolved at the gene level.^3^ Our deepened understanding of the genetic landscape of RP has not only expanded our knowledge but has also paved the way for novel insights into disease mechanisms, sparking cautious optimism concerning the potential for retinal cell rescue and therapeutic interventions.^4^

*RP1*, also known as oxygen‐regulated photoreceptor protein, emerged as the fourth prominent gene within the RP spectrum to be meticulously identified, following the discovery of RHO, RDS, and NRL, which encode rhodopsin, peripherin‐2, and neural retina leucine zipper, respectively.^5^, ^6^, ^7^ Positioned on chromosome 8q12, *RP1* is comprised of four exons, constituting an open reading frame spanning 6468 base pairs. The resultant protein, consisting of 2156 amino acids, is predominantly encoded by exon 4 (788–6468 bp). Intriguingly, previous investigations have unveiled *RP1* mutations as contributors to both autosomal dominant and autosomal recessive forms of RP, perplexing researchers with the dual mutational effect.^8^, ^9^, ^10^

In the current study, we unveil a newfound deletion mutation (c.5326delC, NM_006269.2) located in exon 4 of the *RP1* gene. This mutation was identified in a 19‐year‐old Iranian woman diagnosed with RP‐1, employing a comprehensive approach involving exome‐sequencing and meticulous ophthalmological examination. The integration of advanced genetic sequencing techniques with detailed clinical assessments has provided a nuanced understanding of the molecular intricacies associated with *RP1* mutations, contributing significantly to our comprehension of the genetic underpinnings of RP.

## 2 CASE HISTORY/EXAMINATION

**FIGURE 1:**
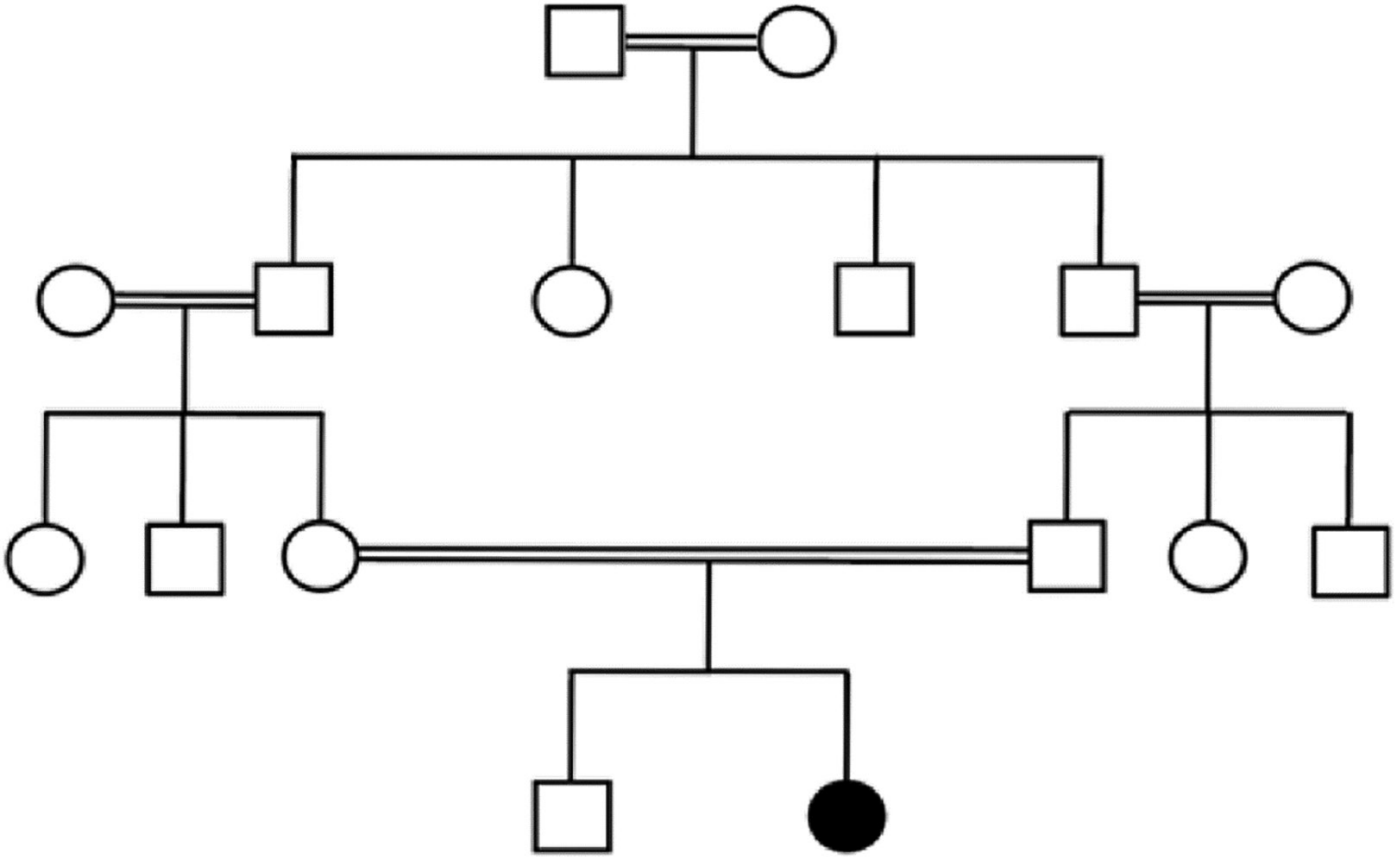
Pedigree of the consanguineous family originating from Iran. The patient is denoted by a distinctive black circle, while circles signify females, and unfilled squares denote males. Consanguinity is visually highlighted through the use of a double horizontal line, providing a clear representation of the familial relationships within the context of this study.

A 19‐year‐old Iranian woman with a 15‐year history of progressive visual impairment sought medical attention. Clinical examination indicated night visual field defects, moderate myopia, and a familial history of consanguinity. Genetic counseling was provided to the patient and her parents, leading to the construction of a familial pedigree for further investigation (Figure 1). Comprehensive information on the medical history was gathered through a thorough review of the patient's medical records.

## 3 METHODS

### 3.1 Differential diagnosis

Considering the presentation of progressive visual impairment, night visual field defects, and familial history, a thorough differential diagnosis was undertaken. Key considerations led to an RP, prompting additional investigations.

### 3.2 Investigations

#### 3.2.1 Exome‐sequencing

Exome‐sequencing was exclusively performed for the proband. Peripheral blood DNA extraction utilized a standard salting‐out method. The patient's DNA was captured using the Agilent SureSelect Human All Exon Kit V6 (Agilent Technologies, Santa Clara, CA, USA) and subsequently sequenced on an Illumina HiSeq 4000 machine (Illumina, San Diego, CA, USA) following the manufacturer's protocols. The average read depth exceeded 100×, with a depth of 20× or greater achieved in 98.0% of the targeted genomic sequence.

#### 3.2.2 Mutation filtering and annotation

Sequenced reads underwent trimming for adaptor sequences and masking for low‐complexity or low‐quality sequencing. Subsequently, reads were mapped to the hg19 whole genome using the mem algorithm from the BWA aligner. Variant calling was executed employing the tools specified in the GATK Best Practices pipeline (https://gatk.broadinstitute.org/hc/en‐us). For comprehensive variant annotation, the ANNOVAR tool was employed.

#### 3.2.3 Mutation validation and co‐segregation analysis

The candidate variant identified through exome‐sequencing was confirmed by Sanger sequencing using specific primers (Forward primer–ATGGACGGCACCCTGGTG and Reverse primer–CTCGCTGGCACTTGGGTC). Sanger sequencing was conducted on an ABI 3130 Genetic Analyzer for accurate validation. Co‐segregation analysis verified the mutation's heterozygous status in the parents and homozygous status in the proband.

#### 3.2.4 Treatment

Currently, there is no curative treatment for RP. Management primarily focuses on symptom alleviation, genetic counseling, and supportive measures, including low‐vision aids and psychological support.

## 4 CONCLUSION AND RESULTS

### 4.1 Outcome

**FIGURE 2:**
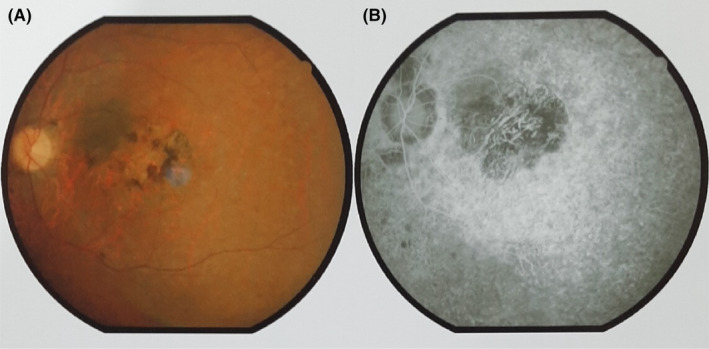
Illustrates bull's‐eye macular atrophy in our patient with RP. (A) This patient showed typical RP changes in the mid‐peripheral region with the superior sector spared. (B) The macular atrophy is apparent with a remaining ring of perifoveal and an island of foveal, forming a bull's‐eye configuration.

Ophthalmic examination revealed severe choroidal and retinal pigment epithelium atrophy, posterior staphyloma, full‐thickness macular hole, and classic fundus manifestations. The patient exhibited features consistent with a diagnosis of RP, including narrow blood vessels, waxy pale optic disc pallor, bone spicules, pigment clumps, attenuated retinal vessels, and cystoid macular edema. A remaining ring of perifoveal and an island of foveal forming a bull's‐eye configuration were also diagnosed (Figure 2).

### 4.2 Genetic analysis

**FIGURE 3:**
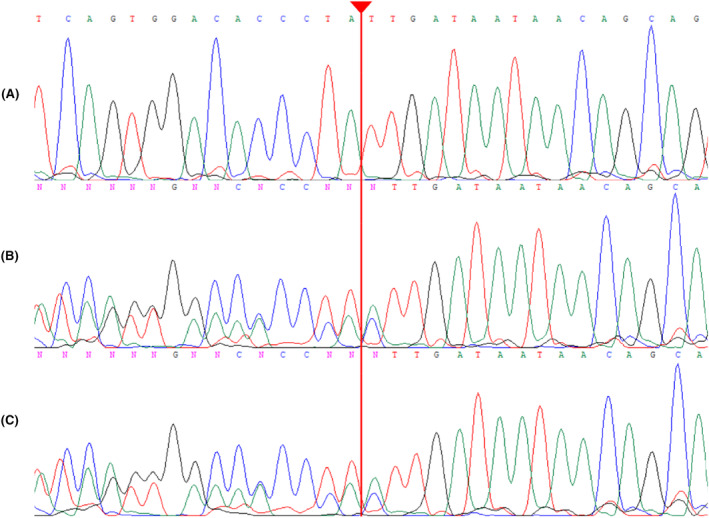
Depicts the identification of a previously unreported mutation in the *RP1* gene and the genetic screening conducted within the studied family. Upon direct sequencing of the patient's DNA (A), a newfound homozygous mutation (c.5326delC) was identified. Furthermore, the genetic analysis of the patient's healthy parents (B, C) demonstrated that they both carry the detected mutation in the heterozygous state.

**FIGURE 4:**
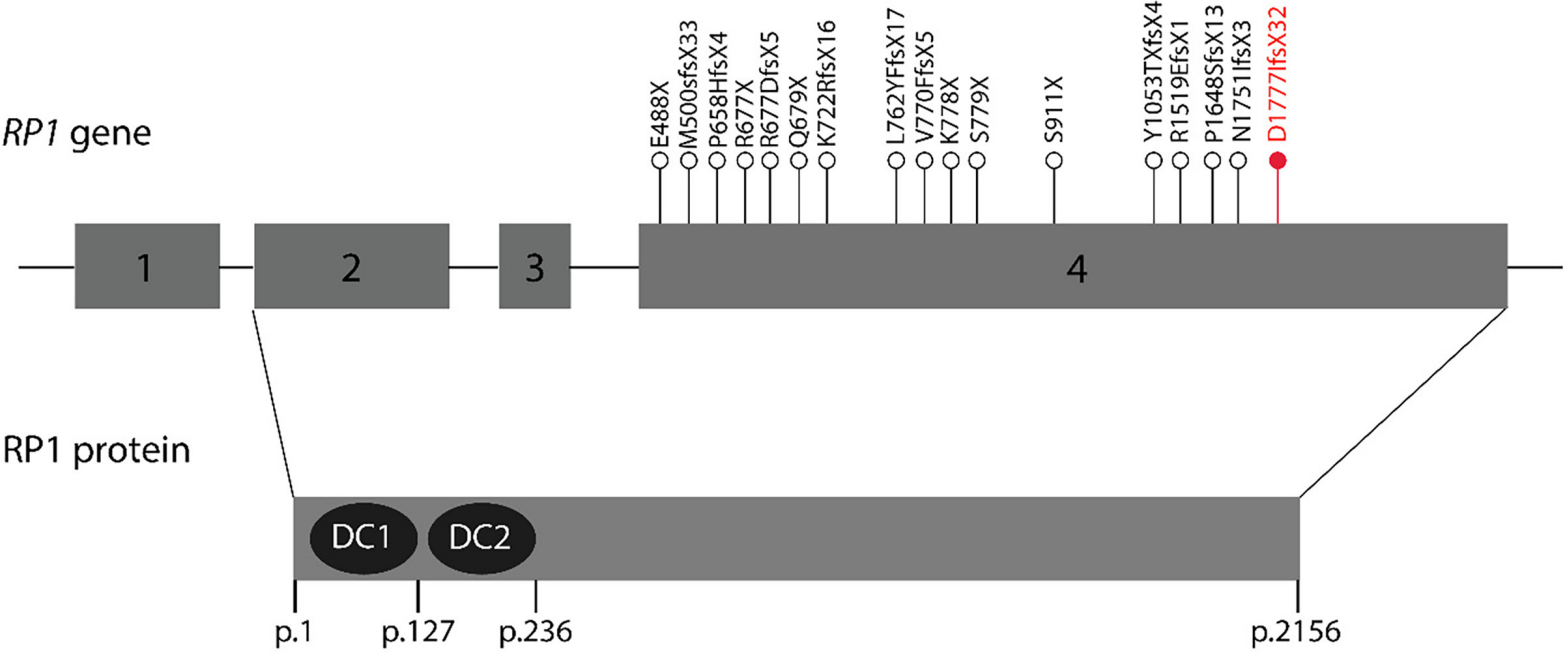
The *RP1* gene structure, highlighting the locations of truncation mutations known to be pathogenic up to the present. The mutations are labeled at the protein level using a reference sequence sourced from Ensembl (ENST00000220676). Pathogenic mutations associated with autosomal dominant or autosomal recessive RP are depicted in black text, while the mutation uncovered in the current study is highlighted in red text. The lower segment of the diagram displays the schematic RP1 protein, featuring the two doublecortin domains encoded by exon 2 (p.29–127) and exon 3 (p.150–236).

The *RP1* gene mutation, NM_006269.2: c.5326delC p.D1777Ifs*32 (GenBank: LC795556.1), identified through exome‐sequencing and confirmed by Sanger sequencing, showed homozygosity in the proband and heterozygosity in the parents. The mutation causes a frameshift leading to a truncated RP1 protein, suggesting its association with RP‐1 progression. Notably, the mutation clusters within exon 4, resulting in a truncated protein lacking approximately one‐third of its total length. Importantly, it does not undergo the nonsense‐mediated decay (NMD) pathway due to its impact on the last exon of the gene.^11^ This mutation, not reported in other RP patients, is visually represented in Figure 3 (Sanger sequencing results) and Figure 4 (mutation clustering within exon 4).

### 4.3 Follow‐up

#### 4.3.1 Genetic counseling for family planning

Given the hereditary nature of *RP1*, ongoing genetic counseling sessions will include discussions about family planning and the potential risk of passing the *RP1* gene mutation to offspring. The patient and her parents will be provided with detailed information regarding genetic testing options for prenatal diagnosis and pre‐implantation genetic testing, empowering them to make informed decisions about future pregnancies.

#### 4.3.2 Prenatal and perinatal guidance

Should her parents decide to pursue pregnancy, specialized prenatal and perinatal care will be recommended. Collaborative efforts with obstetricians and genetic specialists will ensure that the pregnancy is monitored closely, taking into account the potential impact of *RP1* on maternal and fetal health. Timely interventions and support will be provided to address any arising concerns during pregnancy.

#### 4.3.3 Reproductive technologies

For couples considering assisted reproductive technologies, consultations with fertility specialists will be facilitated. These discussions will explore options such as in vitro fertilization (IVF) with pre‐implantation genetic testing to select embryos without the *RP1* mutation before implantation.

#### 4.3.4 Continued psychosocial support

The emotional aspects of managing RP and navigating the complexities of family planning will be acknowledged and addressed through ongoing psychosocial support. Counseling services will be extended to encompass the unique challenges that may arise during the process of planning and experiencing pregnancy, fostering emotional well‐being and resilience.

#### 4.3.5 Research participation and updates

Should the patient express interest, information about relevant research studies or clinical trials exploring advancements in genetic therapies or interventions that may impact the genetic transmission of *RP1* will be shared. Active participation in research initiatives can contribute not only to the patient's own understanding of RP‐1 but also to the broader scientific community's knowledge base.

In conclusion, our study illuminates a distinctive case of RP‐1 in a 19‐year‐old Iranian woman with a previously unreported homozygous pathogenic mutation (c.5326delC) in *RP1* gene exon 4 (NM_006269.2). This mutation is strongly implicated in the observed retinal phenotypes, underscoring the crucial role of precise variant identification for prognosis and genetic consultations. Beyond diagnosis, our follow‐up plan integrates reproductive health considerations into RP‐1 management, supporting the patient and her family through informed decision‐making and personalized care. This patient‐centered approach recognizes the broader impact of RP‐1 on familial dynamics and reproductive choices, ensuring sustained support.

## 5 DISCUSSION

In this study, the affected Iranian woman exhibited a spectrum of symptoms typical of RP, including prolonged history of visual impairment, coupled with night visual field defects and moderate myopia. The unique genetic makeup of the identified mutation adds a layer of complexity to the clinical presentation, emphasizing the importance of genetic testing in establishing an accurate diagnosis. The case report serves as a practical learning point for clinicians, genetic counselors, and researchers, emphasizing the significance of integrating genetic testing into the diagnostic and management pathways for RP‐1. The identification of rare mutations, such as *RP1*:c.5326delC; p.Asp1777Ilefs*32, highlights the need for ongoing collaboration between clinical and research communities to unravel the intricacies of rare genetic disorders.

*RP1*, a gene encoding a protein comprised of 2156 amino acids, exhibits a distinct localization within the connecting cilia of both rod and cone photoreceptors.^12^ The highly specialized expression of *RP1* is predominantly confined to the photoreceptor cells residing in the delicate layers of the retina.^13^, ^14^, ^15^ The uniqueness of *RP1* lies in its N‐terminal region, which exhibits intriguing homology with doublecortin (DCX), a protein initially acknowledged for its role in guiding neuronal migration but subsequently identified as part of an emerging family of microtubule‐associated proteins.^16^, ^17^, ^18^, ^19^ It is noteworthy that *RP1* stands out as the inaugural member of this family to be specifically associated with photoreceptors.^20^ The distinct localization of *RP1* within the connecting cilia, coupled with its structural resemblance to DCX, positions *RP1* as a compelling candidate for orchestrating the intricate transport of newly synthesized outer segment proteins. This transport process unfolds from the inner segments to the critical assembly site of disc membranes, a journey meticulously facilitated through the connecting cilia. A plausible mechanistic insight involves the interaction of *RP1* with microtubules via its N‐terminal DCX domain. Simultaneously, the C‐terminal segment may engage in binding with a protein earmarked for the outer segment. This dynamic interplay suggests a multifaceted role for *RP1* in the orchestration of this intricate transport process. Moreover, beyond its role in protein transport, *RP1* may contribute significantly to the regulation of microtubule dynamics. The inclusion of a DC domain further implies a potential role in maintaining the structural integrity and orientation of the connecting cilia. Additionally, *RP1* might play a crucial role in hindering the unregulated diffusion of substances between the inner and outer segments, thereby contributing to the overall stability and function of the photoreceptor cells.^12^

Mutations within the *RP1* gene play a significant role in the etiology of autosomal dominant RP, accounting for approximately 5.5% of cases, and are also implicated in around 1% of autosomal recessive RP cases.^2^ The literature reports a total of 47 *RP1* mutations linked to RP, with a notable concentration in exon 4 and positioning downstream from the DCX domain, which spans exons 2 and 3. Figure 4 visually represents the distribution of these mutations, with the exception of a recessive mutation (p.A221GfsX20) found in exon 3.^21^ The majority of *RP1* mutations are characterized by single nucleotide substitutions, leading to premature stop codons or frameshift changes resulting from insertions or deletions. Consequently, these mutations give rise to truncated proteins, as depicted in Figure 4.^22^ An intriguing aspect of these mutations is that they occur after the final intron‐exon junction in *RP1*, raising the possibility that the mutant *RP1* transcripts may evade NMD, thereby allowing the production of truncated RP1 proteins. However, it is crucial to note that the empirical confirmation of this potential mechanism is still pending. Significantly, the hypothesis gains support from the detection of mutant *RP1* mRNA in individuals homozygous for a p.Arg677Stop mutation in exon 4.^23^ Despite the seminal report by Khaliq et al. in 2005,^8^ which initially associated *RP1* mutations with autosomal recessive RP, the development of a definitive model distinguishing dominant from recessive mutation mechanisms within *RP1* has proven elusive. Although the concept of haploinsufficiency has been proposed, the perplexing observation of asymptomatic carriers possessing the recessive p.A221GfsX20 mutation, which is predicted to undergo NMD, suggests the existence of alternative mechanisms.^8^ This intriguing observation gains additional support from reports of compound heterozygosity in autosomal recessive RP cases. In such instances, carriers of one allele predicted to undergo NMD also manifest no discernible symptoms, challenging the conventional haploinsufficiency hypothesis.^24^ The protein‐truncating mutation (p.Asp1777IlefsX32) associated with autosomal recessive RP‐1, as discussed in this study, is situated downstream of the DCX domain. Carrier individuals, as depicted in Figure 3, exhibit no apparent signs of a retina phenotype. This observation suggests that each allele, when present in a heterozygous state, does not exert a dominant negative effect, and the presence of one normal allele is adequate for proper retina function. Consequently, it is plausible that crucial protein domains in the C‐terminal region of the RP1 protein are lost in these truncated products (truncations occurring from amino acids 1131 to 1158), leading to RP when homozygously lost. In contrast, mutations causing dominant RP have been identified in more N‐terminal regions of the RP1 protein (residues up to and including amino acid 1053). This implies that mechanisms involving dominant negative effects or toxic gain‐of‐function come into play in cases of dominant RP resulting from RP1 protein mutation.

Multiple reports of autosomal recessive RP associated with *RP1* deficiencies have been documented, consistently noting the commencement of visual impairment within the first 10 years of life. Subsequently, a significant decline in both visual field and acuity is observed during the second or third decade, as evidenced in various studies.^25^, ^26^ Additionally, it is worth noting that consanguinity, the practice of individuals within a family marrying one another, can give rise to the emergence of several homozygous regions in the genetic makeup of offspring.^25^ Similarly, in our case, a 19‐year‐old woman from a consanguineous family, carrying an autosomal recessive form of RP‐1 linked to the *RP1* gene, has been experiencing a slow progression of poor vision. The onset of symptoms commenced approximately 15 years ago, when she was just 4 years old.

Silva RS et al., in 2020, analyzed 529 Brazilian patients suffering from inherited retinal disorders. They revealed six separate *RP1* variants by performing exome sequencing. These results expand the spectrum of *RP1* variants. They reported two pathogenic variants, which were two novel frameshift duplications (c.1265dupC and c.1234dupA), and four familiar pathogenic variants, which were two frameshift deletions (c.3843delT and c.469delG) and two stop gain variants (c.1625C > G and c.1186 C > T).^27^ Similar to our study, they evaluated the fundus features that clinically could prove the assumption of RP‐1 because of the *RP1* gene.

In another study, Riera M et al. 2020, reveia homozygous missense mutation in the *RP1* gene c.606C > A; p.Asp202Glu as the principal gene that causes IRD in 12 families who identify as Kuwaitis. These patients manifested equivalent symptoms since adolescence, including a progressive reduction in visual acuity. Fundus examination showed bilateral macular retinal pigment epithelium disruptions with no perimacular flecks or peripheral alterations. Moreover, three novel *RP1* variants, including nonsense or frameshifting mutations, were detected in three Spanish autosomal recessive RP families and one autosomal dominant RP pedigree.^28^ Similarly, the symptoms in our reported case included visual acuities, defects in the night visual field, and macular atrophy. In our case, ophthalmic examination revealed a significant area of severe choroidal and retinal pigment epithelium atrophy in the macular region related to posterior bowing or posterior staphyloma with a full‐thickness macular hole and a small number of pigment clumps present in the left eye of the patient.

Lastly, our study has brought to light a novel genetic aberration in the form of a 1‐bp deletion mutation (c.5326delC), thereby shedding light on an unexplored facet of the *RP1* truncation mutation. The consequential impact of this mutation is the predicted production of a truncated protein, specifically identified as p.Asp1777IlefsX32. This truncation, in turn, is associated with the onset of RP‐1, highlighting a previously unknown molecular mechanism contributing to the development of this ocular disorder. The exact mechanism by which this mutation leads to RP‐1 may involve the disruption of cellular pathways involved in maintaining retinal health and function. The identification of such intricacies not only advances our understanding of the genetic underpinnings of retinal diseases but also opens avenues for targeted therapeutic interventions and further exploration of the broader implications of these mutations in the realm of genetic disorders.

## AUTHOR CONTRIBUTIONS

**Mostafa Neissi:** Exome data analysis, mutation screening and segregation analysis in the family by Sanger sequencing, and writing – original draft. **Motahareh Sheikh‐Hosseini:** Exome data analysis, and helped to review the manuscript. **Javad Mohammadi‐asl:** Exome data analysis.

## FUNDING INFORMATION

No external funding was received for this study.

## CONFLICT OF INTEREST STATEMENT

The authors declare no conflict of interest.

## CONSENT

The family has granted written informed consent for this publication.

## Data Availability

The data that support the findings of this study are available from the corresponding author upon reasonable request.
